# The case of huge simple pulmonary aspergilloma that responded remarkably to drug therapy

**DOI:** 10.1002/rcr2.1337

**Published:** 2024-03-28

**Authors:** Makoto Yokota, Junji Takiguchi, Hiromi Tomioka

**Affiliations:** ^1^ Respiratory Medicine Kobe City Hospital Organisation, Kobe City Medical Center West Hospital Kobe Japan

**Keywords:** pharmalogical treatment, pulmonary aspergilloma, voriconazole

## Abstract

The first choice of treatment for simple pulmonary aspergilloma is surgery, but in clinical practice, many cases find surgery difficult. We report a case of simple pulmonary aspergilloma in which significant improvement was observed with pharmacological treatment alone, despite initially presenting with a large fungus ball.

## Graphical abstract

We report a case of simple pulmonary aspergilloma accompanied by a large tomour shadow that improved with pharmacological treatment alone.

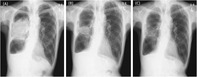

## CLINICAL IMAGE

**FIGURE 1:**
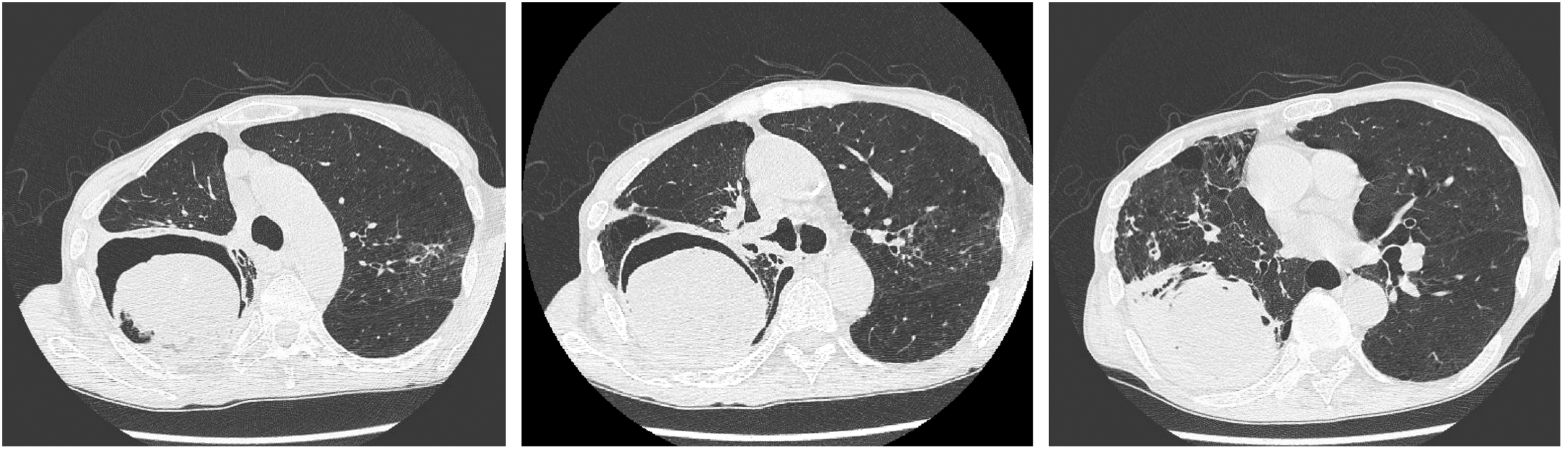
Chest radiographs and computed tomography (CT) scans showed emphysematous changes in the background lungs, and bullae are scattered. A large bulla with a fungus ball in the right upper lobe compresses the middle lobe and lower lobe, with surrounding infiltrative shadows.

**FIGURE 2:**
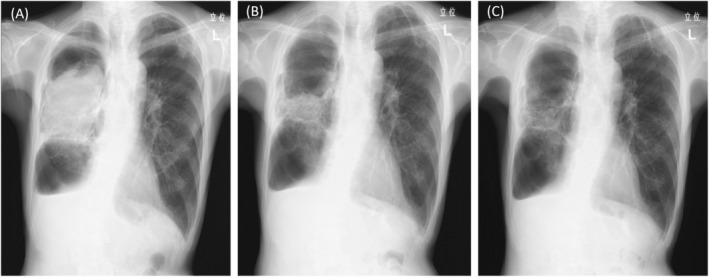
(A) Chest radiograph of anteroposterior view on the first hospital day. (B) Chest radiograph at 5 months after treatment initiation indicated that fugus ball had decreased in size. (C) Chest radiograph at 6 months after treatment initiation indicated that fugus ball had nearly resolved.

A 67‐year‐old male presented to a local clinic with productive cough lasting 1 month. Chest x‐ray examination revealed a large mass‐like opacity in the upper right lung field, prompting referral to our institution. On auscultation, decreased breath sounds were noted in the right upper to middle lung fields. Moreover, serum levels of Aspergillus immunoglobulin G antibody were elevated. Chest computed tomography showed a bulla in the right lung including a large fungus ball (Figure 1 and Figure 2A). Bronchoscopy did not demonstrate gross communication to the fungus ball. Nevertheless, biopsy, brushing, and lavage were performed from the same site under echo guidance, yielding a positive culture for *Aspergillus fumigatus* and a negative culture for acid‐fast bacilli. Oral voriconazole therapy was initiated due to the surgical difficulty arising from the background lung.^1^, ^2^ The treatment resulted in significant reduction and near disappearance of the fungus ball. Complaints of expectoration of black powder in sputum following treatment suggested physical expulsion of the fungal mass rather than solely reduction due to medication. Treatment has been continued with intermittent pauses, with the shadow remaining reduced. We observed significant improvement in a large fungal ball (i.e., simple pulmonary aspergilloma) through pharmacological treatment alone (Figures 2B and C).

## CONFLICT OF INTEREST STATEMENT

None declared.

## ETHICS STATEMENT

The authors declare that appropriate written informed consent was obtained for the publication of this manuscript and accompanying images.

## Data Availability

Data sharing not applicable—no new data generated.
